# Leukocyte Membrane-Coated Liquid Metal Nanoswimmers for Actively Targeted Delivery and Synergistic Chemophotothermal Therapy

**DOI:** 10.34133/2020/3676954

**Published:** 2020-06-24

**Authors:** Daolin Wang, Changyong Gao, Chang Zhou, Zhihua Lin, Qiang He

**Affiliations:** Key Laboratory of Microsystems and Microstructures Manufacturing, Ministry of Education, Micro/Nanotechnology Research Centre, Harbin Institute of Technology, 92 West Dazhi Street, Harbin 150080, China

## Abstract

We report a leukocyte membrane-coated gallium nanoswimmer (LMGNS) capable of ultrasound-propelled motion, antibiofouling, and cancer cell recognition and targeting. The LMGNS consists of a needle-shaped gallium core encapsulating an anticancer drug and a natural leukocyte membrane shell. Under the propulsion of an ultrasound field, LMGNSs could autonomously move in biological media with a speed up to 108.7 *μ*m s^−1^. The velocity and motion direction of the LMGNSs can be modulated by regulating the frequency and voltage of the applied ultrasound field. Owing to the leukocyte membrane coating, LMGNSs can not only avoid biofouling during the motion in blood but also possess cancer cell recognition capability. These LMGNSs could actively seek, penetrate, and internalize into the cancer cells and achieve enhanced anticancer efficiency by combined photothermal and chemical therapy. Such biofunctionalized liquid metal nanoswimmer presents a new type of multifunctional platform for biomedical applications.

## 1. Introduction

Nanoswimmers, which convert diverse types of energy into mechanical movement [1–5], hold great promise in providing solutions to various future technological needs, such as controlled capture [6, 7], electronic repair [8], environmental remediation [9, 10], and complex microstructure fabrication [11, 12]. In particular, synthetic nanoswimmers may perform diverse operations in the biomedical field, including biosensing [13, 14], diagnostics [15, 16], precision surgery [17], laser tissue welding [18], and direct drug delivery [19, 20]. In recent years, various strategies have been employed to propel such micro/nanoswimmers, including chemical reaction [21, 22], light stimuli [23–25], and electric [26, 27], magnetism [28, 29], and acoustic [30, 31] actuation. However, most of the nanoswimmers still rely on inorganic materials, such as SiO_2_, mesoporous silicon, and Fe_3_O_4_; these formulations often fail to be useable in the biosystem for the systemic toxicity or poor biodegradability.

Liquid metal, a biofriendly material with excellent properties including low melting point, large surface tension, and high thermal and electric conductivity [32–34], has emerged for stretchable electronics and soft robots [35–39]. In our previous work [40], we demonstrated a first example of the soft rod-like liquid metal nanoswimmers which could transform from rod to droplet, fuse together, and be degraded in both an acidic buffer and biomedium of cancer cells. To date, gallium-based liquid metal has aroused great interest in the field of biomedicine [41–43]. However, it is still a challenge to fabricate liquid metal-based nanoswimmers with the capability of active motion, antibiofouling, and targeted drug delivery.

**Scheme 1:**
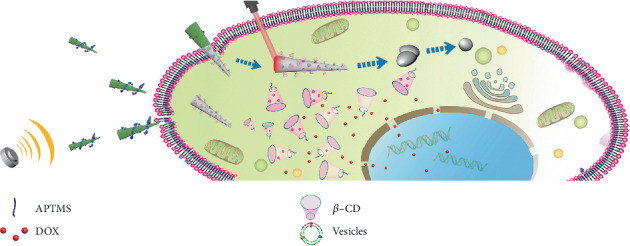
The acoustically propelled leukocyte membrane-coated gallium nanoswimmers (LMGNSs) actively target, penetrate, and kill the cancer cell.

Here, we demonstrate an acoustically propelled leukocyte membrane-coated gallium nanoswimmer (LMGNS), which can be served as a multifunctional platform for precise cancer therapy, as shown in Scheme 1. The biomimetic nanoswimmers were fabricated by combining the pressure-filter-template method [40] and cell membrane-cloaking technique. The as-fabricated LMGNS has a needle-like structure, thus displaying autonomous motion under the propulsion of the ultrasonic field. The velocity and direction of the LMGNSs can be regulated by alternating frequency and voltage of the ultrasound field. Moreover, the LMGNSs have the capacities of antibiofouling, prolonged motion in biological medium, and recognition of cancer cells. LMGNSs could also actively target and penetrate the predefined cancer cells. With the combination of photothermal therapy and targeted drug delivery, LMGNSs display enhanced anticancer efficacy.

## 2. Results and Discussion

**Figure 1:**
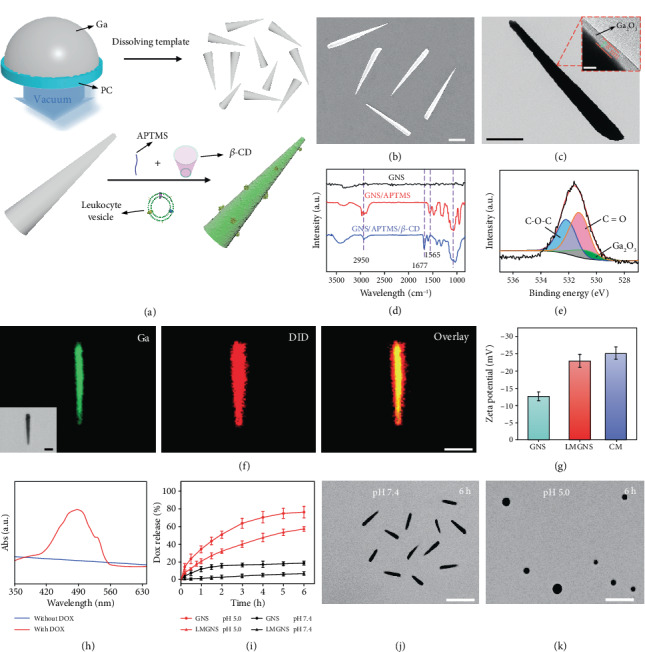
The characterization of LMGNSs. (a) Schematic illustration of the fabrication process of leukocyte membrane-coated gallium nanoswimmers (LMGNSs). (b) SEM image of GNSs. Scale bar, 2 *μ*m. (c) TEM images of GNS, scale bar 2 *μ*m, the inset is an enlarged TEM image of the red region showing a core-shell structure, scale bar 10 nm. (d) The infrared spectra of GNSs before and after modification. (e) The O 1s XPS spectrum of the GNSs. (f) The CLSM images of the LMGNS, scale bars 2 *μ*m. (g) Zeta potential of GNSs, LMGNSs, and cell membrane vesicles (CM). (h) The UV-vis spectra of the LMGNSs before and after loading Dox. (i) The Dox release plots of the GNSs and LMGNSs in different pH conditions. The microscopic images of the Dox-loaded LMGNSs after incubation in pH 7.4 (j) and 5.0 (k) solution for 6 h, respectively, scale bars 10 *μ*m.

As demonstrated in Figure 1(a), to synthesize the leukocyte membrane-coated gallium nanoswimmers (LMGNSs), gallium nanoswimmers (GNSs) were firstly fabricated by using the previously reported pressure-filter-template method [40]. The scanning electron microscopy (SEM) image shows that the obtained GNSs displayed a needle-like shape, with a length of 7.03 ± 0.60 *μ*m and a diameter of 800 ± 51 nm and 153 ± 37 nm at each end (Figure 1(b)). The transmission electron microscopy (TEM) image demonstrates that GNS has a core-shell structure, and the thickness of the shell was about 8 nm (Figure 1(c)). EDX mapping analysis shows that the GNSs were composed by the gallium and oxygen elements, indicating that the shell was Ga_2_O_3_ (Figure S1). In order to load the anticancer drug, aminopropyltrimethoxysilane (APTMS) and carbonylated *β*-cyclodextrin (*β*-CD) were modified onto the shell of GNSs. After modification, Fourier transform infrared (FTIR) spectra show that GNSs presented new characteristic peaks at 2950 cm^−1^, 1565 cm^−1^, 1000 to 1100 cm^−1^, and 1677 cm^−1^, which may correspond to the stretching vibration of C-H, the bending vibration of N-H, the Si-O bond from APTMS, and the stretching vibration of the C=O group from carbonylated *β*-CD, respectively (Figure 1(d)). The X-ray photoelectron spectroscopy (XPS) spectrum in Figure 1(e) shows the three subcomponent peaks of O 1s with binding energies at 530.8 eV (Ga_2_O_3_), 531.2 eV (C=O), and 532.1 eV (C-O-C). And Figure S2 demonstrates the two states of nitrogen at 398.7 eV (C-N) and 399.7 eV (C-N-C). These results suggest that aminopropyltrimethoxysilane and carbonylated *β*-CD were successfully modified onto the surface of GNSs.

After that, leukocyte membrane vesicles were isolated through a physical extrusion procedure [44] and then were fused onto the surface of GNSs through an acoustically assisted nanovesicle fusion method, as shown in Figure S3. Then, the structure of the LMGNSs was studied by using confocal laser scanning microscopy (CLSM), in which the leukocyte membranes were labeled with 1,1'-dioctadecyl-3,3,3',3'-tetramethylindodicarbocyanine (DiD) before fusion. CLSM images show that the green fluorescence displayed a high degree of colocalization with the red fluorescent signals (Figure 1(f)). It can be confirmed that the green fluorescence excited by a 488 nm light came from GNSs. However, the source of the red fluorescence is uncertain, because the GNSs also could display red photoluminescence under the irradiation of a certain intensity of 633 nm light [40]. Thus, we analyzed the CLSM images of GNSs and LMGNSs excited by 633 nm light at different intensities. As shown in Figure S4, only the LMGNSs showed noticeable fluorescence at the same laser intensity as in Figure 1(f) (4 mV), indicating that the red fluorescence came from the DiD-labeled leukocyte membrane. We also found that the zeta potential changed from -13.1 mV (GNSs) to -22.9 mV (LMGNSs), which is similar to the value of a leukocyte membrane (Figure 1(g)). Taken together, these results demonstrate that leukocyte membrane vesicles fused onto the surface of GNSs and LMGNSs were successfully fabricated.

Then, these LMGNSs were explored to transport anticancer drugs, and their drug-loading capacity was evaluated, in which doxorubicin (Dox) was chosen as a model drug. After being loaded with Dox, a characteristic absorption peak of Dox at 480 nm was found, indicating the successful encapsulation of Dox (Figure 1(h)). The Dox-loading rate was determined by measuring the mass of nanoswimmers before and after Dox encapsulation. We found that the mass of LMGNSs before and after encapsulation was 5.3 and 6.1 mg, respectively, which indicates that the Dox-loading capacity was about 13.1%wt. The Dox release profiles of GNSs-Dox and LMGNSs-Dox were evaluated through the dialysis method in the PBS buffer with the pH of 5.0 and 7.4. The released Dox from GNSs-Dox and LMGNSs-Dox in the acidic buffer was 47.4% and 72.6% within 4 h, respectively; however, there were only 5.3% and 17.7% in the neutral buffer, respectively (Figure 1(i)). The differences in released amount between GNSs-Dox and LMGNSs-Dox at both acidic and neutral conditions may be attributed to the cloaked membrane. These results indicate that LMGNSs could act as a pH-sensitive cargo for drug delivery. The LMGNSs could maintain their original shape for 6 h in the neutral medium (Figure 1(j)). When exposed to acidic media, they transformed and fused with each other (Figure 1(k)).

**Figure 2:**
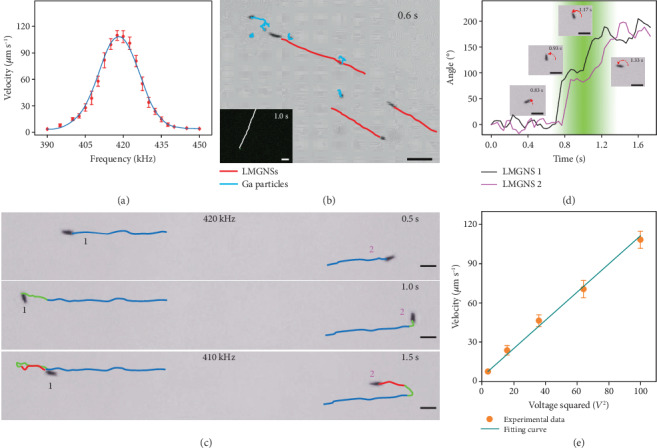
The acoustically propelled motion of the LMGNSs. (a) The relationship between the motion velocity of LMGNSs and frequency of acoustic field at 10 V. (b) Time-lapse image of a LMGNS at an applied voltage of 10 V and an ultrasound frequency of 420 kHz. Inset is fluorescent time-lapse image of a LMGNS under same condition. (c) The time-lapse images of frequency determined direction control of LMGNSs. (d) The direction changes of LMGNSs during frequency changing. The inset images show the LMGNS 2 at 0.83, 0.93, 1.17, and 1.33 s. (e) The relationship between motion velocity LMGNSs and the applied voltage of acoustic field. Scale bars, 20 *μ*m.

Due to the asymmetric structure, the LMGNSs can be actuated by the ultrasonic field.  Figure 2(a) illustrates the relationship between the speed of the LMGNSs and the ultrasonic frequency with an applied 10 V ultrasound field. The velocity of the LMGNSs increased with the frequency increasing from 390 to 417.5 kHz and then decreased as the frequency increased further. Under the frequency ranging from 415 to 425 kHz, the average velocity of LMGNSs could reach over 100 *μ*m s^−1^.  Figure 2(b) and Video S1 show that the LMGNSs moved approximately in a straight line with an average velocity of 108.8 ± 7.8 *μ*m s^−1^ (15.5 ± 1.1 body s^−1^) under the ultrasound field with the voltage of 10 V and the frequency of 420 kHz. However, the velocity of the tracer gallium nanospheres was only 4.1 ± 0.5 *μ*m s^−1^. These results indicate that the LMGNSs are driven by the primary acoustic radiation force. Owing to their photoluminescence property, the motion of LMGNSs also can be monitored in real time by florescence imaging (Video S2). This is of great significance for the *in vivo* biomedical applications of nanoswimmers.

The ultrasonic frequency can not only regulate the velocity of the LMGNSs but also change their motion direction. As shown in Figure 2(c) and Video S3, the two LMGNSs moved away from each other in opposite directions with an average velocity of 103.5 *μ*m s^−1^ under an ultrasound field 10 V, 420 kHz. The velocity of the LMGNSs then dropped dramatically as the frequency shifts gradually in 1 s. Once the frequency reached 410 kHz, both LMGNSs start to move in reverse directions at a stable velocity of 33.7 *μ*m s^−1^.  Figure 2(d) shows the direction change of the LMGNSs in Figure 2(c), with a reference direction taken at 0 s. The direction fluctuated with little changes under both frequencies due to the effect of Brownian motion and changed quickly as the frequency shifted at 0.7 s, resulting in a dramatic rotation of 180° for both LMGNSs at 1.3 s. The inset microscopic images show the rotating process of LMGNS 2 at times 0.83, 0.93, 1.17, and 1.33 s. These results are consistent with previous theories that the shift of frequency leads to a change in the distribution of acoustic pressure [45].There was a high acoustic pressure node between the two particles which propelled the LMGNSs to move away from each other at 420 kHz. As the frequency shifted, the acoustic pressure distribution gradually changed and a low acoustic pressure node formed at 410 kHz, driving the two LMGNSs close together. We also found that the motion velocity of LMGNSs could be regulated by adjusting the ultrasonic voltage. There was a linear relationship between the velocity and the ultrasonic voltage square *V*^2^ (Figure 2(e)).

**Figure 3:**
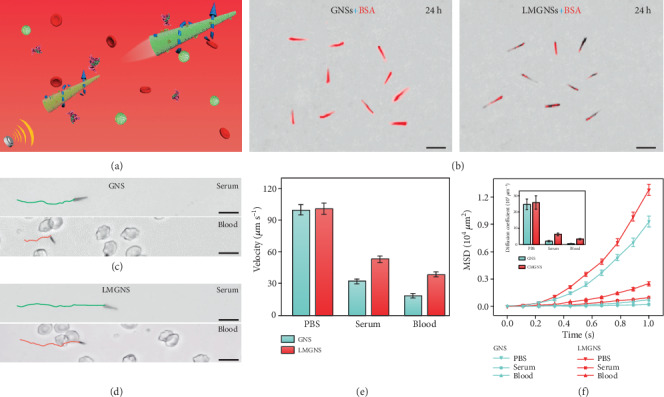
Kinematic analysis of the GNSs and LMGNSs in biological media. (a) Schematic illustration of the acoustically propelled motion of LMGNSs in blood. (b) The CLSM images of GNSs and LMGNSs after cocultured with Rhodamine-labeled BSA for 24 h. The time-lapse images of (c) GNS and (d) LMGNS in the serum and blood under the 420 kHz and 10 V. (e) The velocity of GNSs and LMGNSs in PBS, serum, and blood media. (f) The mean squared displacement (MSD) and diffusion coefficient of the GNSs and LMGNSs in the different solution. All scale bars, 10 *μ*m.

One of the challenges of synthetic nanoswimmers in biomedical applications is the biofouling effect, which not only increases the viscous resistance of the nanoswimmers but also induces immune clearance responses [46]. It is well known that leukocytes are natural immune cells that could circulate in the blood for a long time and recognize cancer cells by proteins on their cell membranes [47]. Therefore, we expect that the leukocyte membrane-coated gallium nanoswimmers (LMGNSs) could possess the antibiofouling function of leukocytes (Figure 3(a)). To evaluate their antibiofouling capacity, the GNSs and LMGNSs were respectively incubated with Rhodamine-labeled bovine serum albumin (Rhodamine-BSA). After incubation for 24 h, the CLSM images show that the red florescence signal of GNSs was much higher than that of LMGNSs, indicating less Rhodamine-BSA was attached to the surface of LMGNSs (Figure 3(b)). For biomedical applications, it is critical to test the propulsion performance of the nanoswimmers in biological environments. Thus, the motion behaviors of the GNSs and LMGNSs in the biological fluids (serum and blood) were tested after being incubated in these media for 24 h. As shown in Figures 3(c) and 3(d) and corresponding Video S4, S5, the movement distances of LMGNSs in the serum and blood were longer than that of GNSs within the same time. The average speed of the GNSs and LMGNSs in different biological media is shown in Figure 3(e). It can be found that the speed of LMGNSs in the serum and blood was 52.9 *μ*m s^−1^ and 35.6 *μ*m s^−1^, respectively, which is significantly higher than that of GNSs (31.9 *μ*m s^−1^ and 20.2 *μ*m s^−1^, respectively). The mean squared displacement (MSD) and the diffusion coefficient (D) show that LMGNSs also displayed a higher diffusion coefficient under the same condition (Figure 3(f)). These results indicate that LMGNSs have good capacity to resist biological fouling and can move in biological medium for a long time.

**Figure 4:**
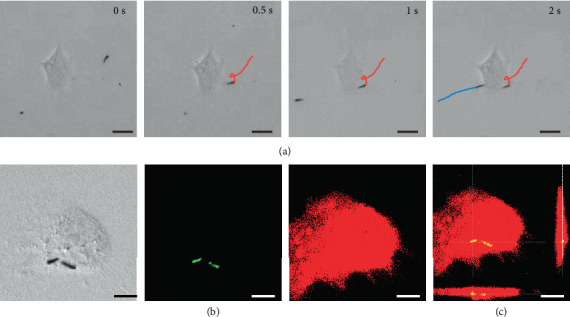
LMGNSs actively recognize, target, and penetrate HeLa cell. (a) Time-lapse images of acoustically propelled LMGNSs actively seeking and targeting the HeLa cell. Scale bars, 20 *μ*m. (b) CLSM images illustrating the internalization of the LMGNSs in the HeLa cell. Scale bars, 10 *μ*m. (c) The 3D reconstruction CLSM image in (b). Scale bar, 10 *μ*m.

The leukocyte membrane coating enables LMGNSs to actively recognize cancer cells in a neutrophil-like way [48]; therefore, LMGNSs were explored in cancer cell-targeted therapy. Firstly, the capacity of LMGNSs for actively recognizing, seeking, and penetrating into the treated cancer cell was evaluated. As shown in Figure 4(a) and the corresponding Video S6, LMGNSs were able to recognize the HeLa cell during the ultrasound field-propelled motion. The attachment to the Hela cell and spinning behavior of the LMGNSs were followed by the penetration and internalization into the cell. CLSM images in Figure 4(b) illustrate the position of the LMGNSs relative to the HeLa cell. The LMGNSs (green) are visualized due to the photoluminescence property of the liquid metal gallium in the 488 nm channel with a laser intensity of 4 mV; meanwhile, DiD was used to label the membrane of the HeLa cell (red). The 3D reconstruction image further demonstrates the intracellular localization of the LMGNSs inside the HeLa cell (Figure 4(c)).

**Figure 5:**
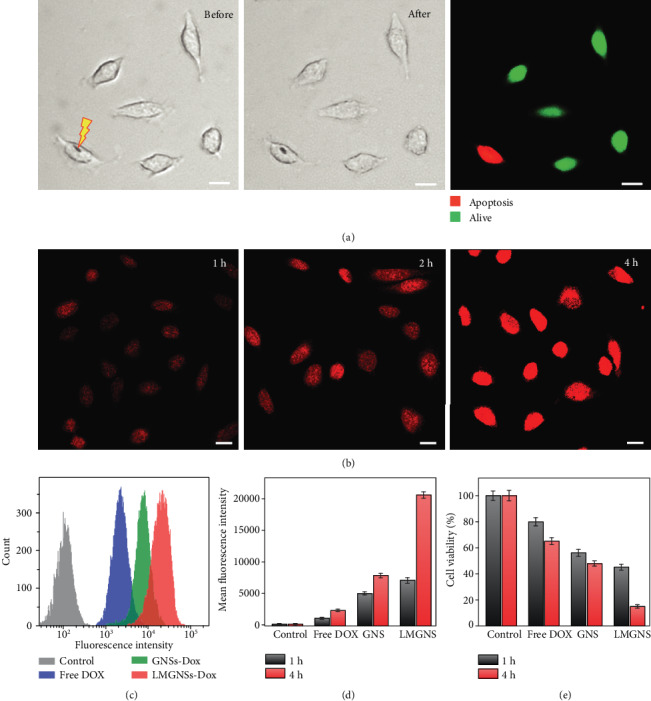
The intracellular photothermal therapy and Dox release of the LMGNSs. (a) Microscopic images of HeLa cells with the LMGNSs before and after NIR laser irradiation and the fluorescence image of HeLa cells after NIR irradiation. (b) CLSM images of the HeLa cells treated with LMGNSs-Dox for 1, 2, and 4 h. (c) Quantitative Dox release analysis of the free Dox, GNS-Dox, and LMGNS-Dox treatments for 4 h by flow cytometry. (d) Mean fluorescence intensity of Hela cells incubated with PBS, free Dox, GNSs-Dox, and LMGNSs-Dox for 1 and 4 h. (e) Therapeutic efficiency of free Dox, GNSs-Dox, and LMGNSs-Dox to HeLa. All scale bars, 20 *μ*m.

The biomedical application potential of LMGNSs was then investigated. The LMGNSs have a high absorption in the near-infrared region, which enables their utilization as a photothermal therapeutic agent [49, 50]. As Figure 5(a) shows, a shape transformation was clearly observed after irradiated by an 808 nm laser at a power of 15 mW·*μ*m^−2^ for 5 s, and the cell viability was indicated by calcein acetoxymethyl (Calcein-AM) and propidium iodide (PI). After the laser radiation, the HeLa cell treated by LMGNSs appeared as red fluorescence which confirmed the necrosis of the cell, while the untreated cells kept alive were indicated by the green fluorescence. The capacity of drug transport and release of LMGNSs were then studied. Fluorescent images in Figure 5(b) exhibit the HeLa cell treated with Dox-loaded LMGNSs for 1, 2, and 4 h. We also found that LMGNS-Dox-incubated HeLa cells displayed higher fluorescence intensity than the GNS-Dox-treated group (Figure S5), which indicates that high cancer uptake of LMGNSs-Dox was attributed to the cell membrane camouflaging. It was found that fluorescence intensity of HeLa cells increased by prolonging the incubation time. The cellular uptake amount of free Dox, GNSs-Dox and LMGNSs-Dox was quantitatively evaluated by using flow cytometry. There was a noticeable peak right shift in the LMGNS-Dox-incubated HeLa cell group (Figures 5(c) and S6, relatively). As shown in Figure 5(d), the mean fluorescence intensity result indicates that the cellular uptake to LMGNSs-Dox was increased to 2.6 times by the leukocyte membrane coated after 4 h incubation, further indicating that the cell membranes cloaked with GNSs increased the cancer target efficiency. We further quantified the efficacy difference of these treatments. MTT assay (Figure 5(e)) shows that only 15% HeLa cells survived after the incubation with the LMGNSs-Dox for 4 h. Compared to free Dox and GNS-Dox treatments, LMGSN-Dox treatment displays higher therapeutic efficiency toward cancer cells.

## 3. Conclusion

We have introduced an acoustically propelled leukocyte membrane-coated gallium nanoswimmer for enhanced photothermal and chemical cancer therapy. The asymmetric needle-like structure and high density allow for powerful propulsion of the LMGNSs. The motion velocity and direction of LMGNSs could be controlled by the voltage and frequency of the applied acoustic field. The integration of the leukocyte membrane enables the LMGNSs with prolonged motion time due to the capability of antibiofouling in the biological medium. Owing to their strong absorptive capacity in the NIR region, excellent drug loading, and pH-responsive release capacity, LMGNSs exhibit a combined photothermal and chemotherapy ability for cancer cells. This LMGNS integrates the capacities of active motion, antibiofouling, cancer cell recognition, imaging, drug delivery, and photothermal cancer treatment, representing a state-of-the-art multifunctional nanoswimmer for the next-generation precision theranostics.

## 4. Materials and Methods

### 4.1. Materials

Gallium, aminopropyltrimethoxysilane (APTMS), and carbonylated *β*-cyclodextrin (*β*-CD) were purchased from Aladdin. Park Memorial Institute 1640 (RPMI 1640) medium, 1,1′-dioctadecyl-3,3,3′,3′-tetramethylindo-dicarbocyanine perchlorate (DiD), dimethyl sulphoxide (DMSO), fetal bovine serum (FBS), 3-(4, 5-dimethylthiazol-2-yl)-2,5-diphenyltetrazolium bromide solution (MTT), penicillin streptomycin, and 0.25% Trypsin-EDTA were obtained from Life Technologies Corporation; ceramic transducers (H4P101000, *ϕ*10∗2 mm) were purchased from Fukeda.

### 4.2. Preparation of GNSs

To synthesize GNSs, the monodispersed needle-like liquid metal nanoparticles were firstly prepared by a previous report [40]. Then, the as-prepared liquid metal nanoparticles in 0.5 mL ethanol were introduced into 0.5 mL ethanol solution with 10 *μ*L APTMS with ultrasonic treatment in an ice bath. The APTMS-modified liquid metal nanoparticles were washed using ethanol and translated to the water solution; subsequently, 80 mg carbonylated *β*-CD was added with continuous stirring for 24 h in an ice bath. The resulting GNSs were obtained after removing the excess carbonylated *β*-CD.

### 4.3. Synthesis of LMGNSs

Leukocyte cells (THP-1) were firstly suspended in a hypotonic dissolution buffer consisting of 0.2 mM EDTA, 1 mm NaHCO_3_, and 1 mm PMSF. After treatment at 4°C overnight, these cells were enucleated for 20 times by using a hand-held Dounce homogenizer. After the above suspension was centrifuged at 3200 g at 4° C for 5 min, the leukocyte membrane precipitate was obtained after centrifugation at 150000 g at 4° C for 1 h. Subsequently, the isolated leukocyte membranes were physically extruded through 1 *μ*m, 400 nm, 200 nm, 100 nm, and 50 nm polycarbonate membranes for 21 passes. The LMGNSs were then fabricated by fusing the leukocyte membrane nanovesicles onto the surface of GNSs following a published protocol [51]. Briefly, the GNSs and leukocyte nanovesicles were mixed and then sonicated by using an Autoscience AS7240AT ultrasonic machine for 2 h in an ice bath. After removing the excess leukocyte membrane nanovesicles, LMGNSs were obtained.

### 4.4. Acoustic Experiments

The acoustic-propelled motion of the LMGNSs was conducted by using an ultrasonic setup, which is made of a function generator (Tektronix AFG1062), signal amplifier (Toellner TOE 7607), and ceramic transducer. And the sample cell is a cylinder with a height of 1.5 mm and a diameter of 5 mm, and the ceramic transducer was pasted on the bottom of the sample cell. The LMGNSs were incubated in the biological media (water, PBS, serum, and blood) for 24 h, and then a 15 *μ*L LMGNS solution was dropped into the sample cell and covered by a square coverslip. A frequency of 390-450 kHz and 0-10 V voltage were applied to actuate the LMGNSs, which was observed by an Olympus optical microscope (OLYMPUS BX53).

### 4.5. Photothermal Treatment Experiments

The photothermal therapy of LMGNSs was observed by the fluorescence microscope (Olympus IX 71). The HeLa cells with LMGNSs were irradiated by an 808 nm laser at the power of 15 mW·*μ*m^−2^ for 5 s. Then, the cell viability is determined by adding PI and Calcein-AM. Finally, the resulting fluorescence image was captured under a 488 nm light.

### 4.6. Flow Cytometry Analysis

The cellular uptake of the various drug-loaded nanoswimmers was evaluated by using the flow cytometry analysis. GNSs-Dox, LMGNSs-Dox, and free Dox with a drug concentration of 10 *μ*g mL^−1^ was cocultured with HeLa cells with for 1 h and 4 h, respectively. After that, the free Dox and nanoswimmers were removed by washing with PBS solution for 5 times. Then, HeLa cells were harvested, and their fluorescence intensity was tested by flow cytometry (BD FACSAria).

### 4.7. MTT Assay

To evaluate their anticancer efficiencies, PBS, free Dox, GNSs-Dox and LMGNSs-Dox first cocultured with HeLa cells for 1 h and 4 h. Then, a drop of 20 mL MTT solution with the concentration of 5 mg mL^−1^ was added into the cell culture medium and incubated with HeLa cells for 4 h. After removing the culture medium, 150 mL DMSO was dropped to HeLa cells, and the cell viability of HeLa cells was measured at the absorbance wavelength of 570 nm by using a microplate reader.

## Supplementary Materials

Supplementary 1 Figure S1: SEM and EDX mapping images of the GNSs. Figure S2: N 1s XPS spectrum of the GNSs. Figure S3: the schematic depicting the preparation process of LMGNSs. Figure S4: CLSM images of GNS and LMGNS excited by a 633 nm light with different intensity. Figure S5: CLSM images of the HeLa cells treated with Dox-loaded GNSs for 1, 2, and 4 h. Figure S6: Quantitative Dox release analysis after treatments for 1 h by flow cytometry.

Supplementary 2 Movie S1: the movement of LMGNSs under 420 kHz, 10 V ultrasound field.

Supplementary 3 Movie S2: the fluorescence movement of LMGNS under 420 kHz, 10 V ultrasound field.

Supplementary 4 Movie S3: the diverging motion of LMGNSs by regulating the ultrasonic frequency.

Supplementary 5 Movie S4: the movement of GNS in the serum and blood solution.

Supplementary 6 Movie S5: the movement of LMGNS in the serum and blood solution.

Supplementary 7 Movie S6: the movement of LMGNSs actively target the HeLa cell.
