# Supplementary material for: Leukocyte Membrane-Coated Liquid Metal Nanoswimmers for Actively Targeted Delivery and Synergistic Chemophotothermal Therapy
Source: Research (Wash D C). 2020 Jun 24;2020:3676954. doi: 10.34133/2020/3676954 (PMC7333182; doi:10.34133/2020/3676954)
Supplement: Supplementary 1 — Figure S1: SEM and EDX mapping images of the GNSs. Figure S2: N 1s XPS spectrum of the GNSs. Figure S3: the schematic depicting the preparation process of LMGNSs. Figure S4: CLSM images of GNS and LMGNS excited by a 633 nm light with different intensity. Figure S5: CLSM images of the HeLa cells treated with Dox-loaded GNSs for 1, 2, and 4 h. Figure S6: Quantitative Dox release analysis after treatments for 1 h by flow cytometry. [file 3676954.f1.doc]

***Supporting Information***


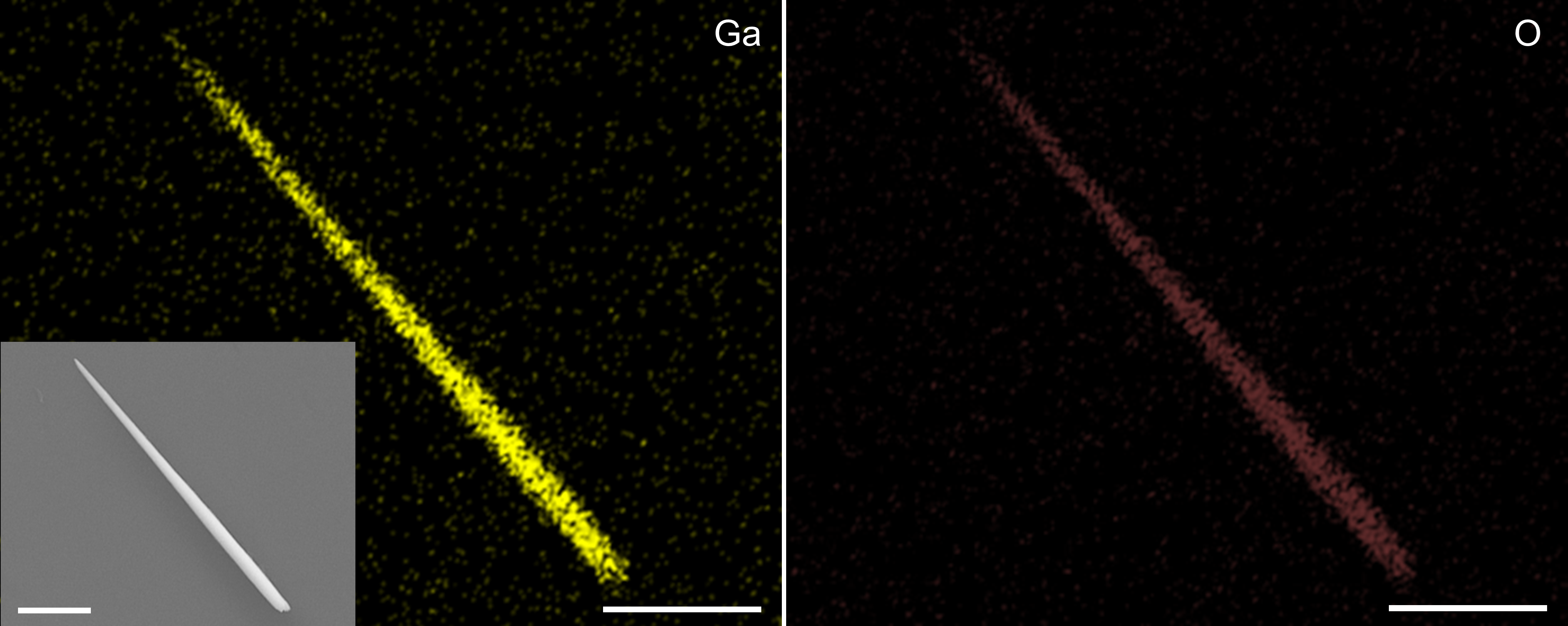


**Figure S1.** The SEM and EDX mapping images of the liquid metal gallium nanoswimmer. Scale bar, 2 μm.


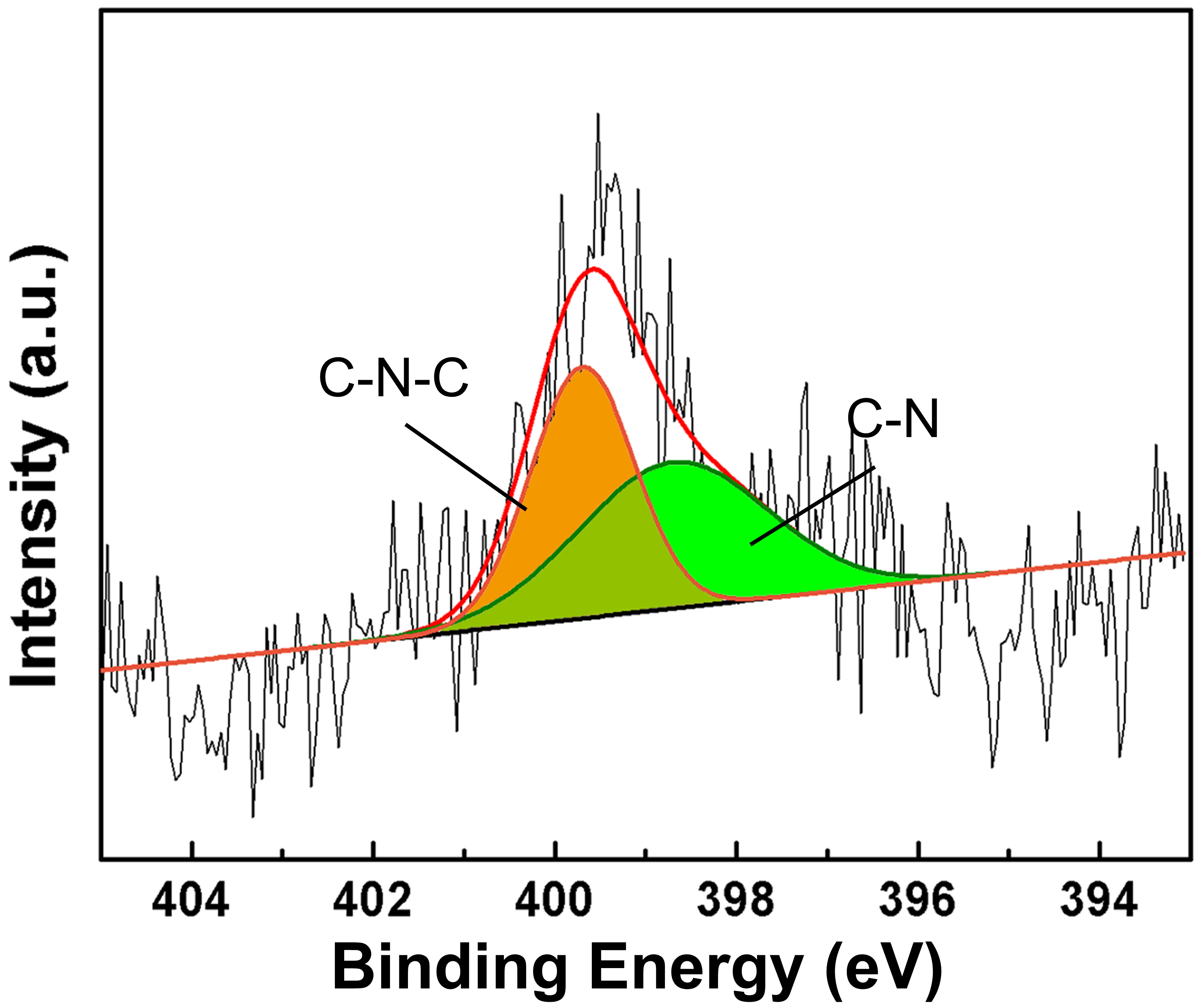


**Figure S2.** The N 1s XPS spectrum of the liquid metal gallium nanoswimmers.


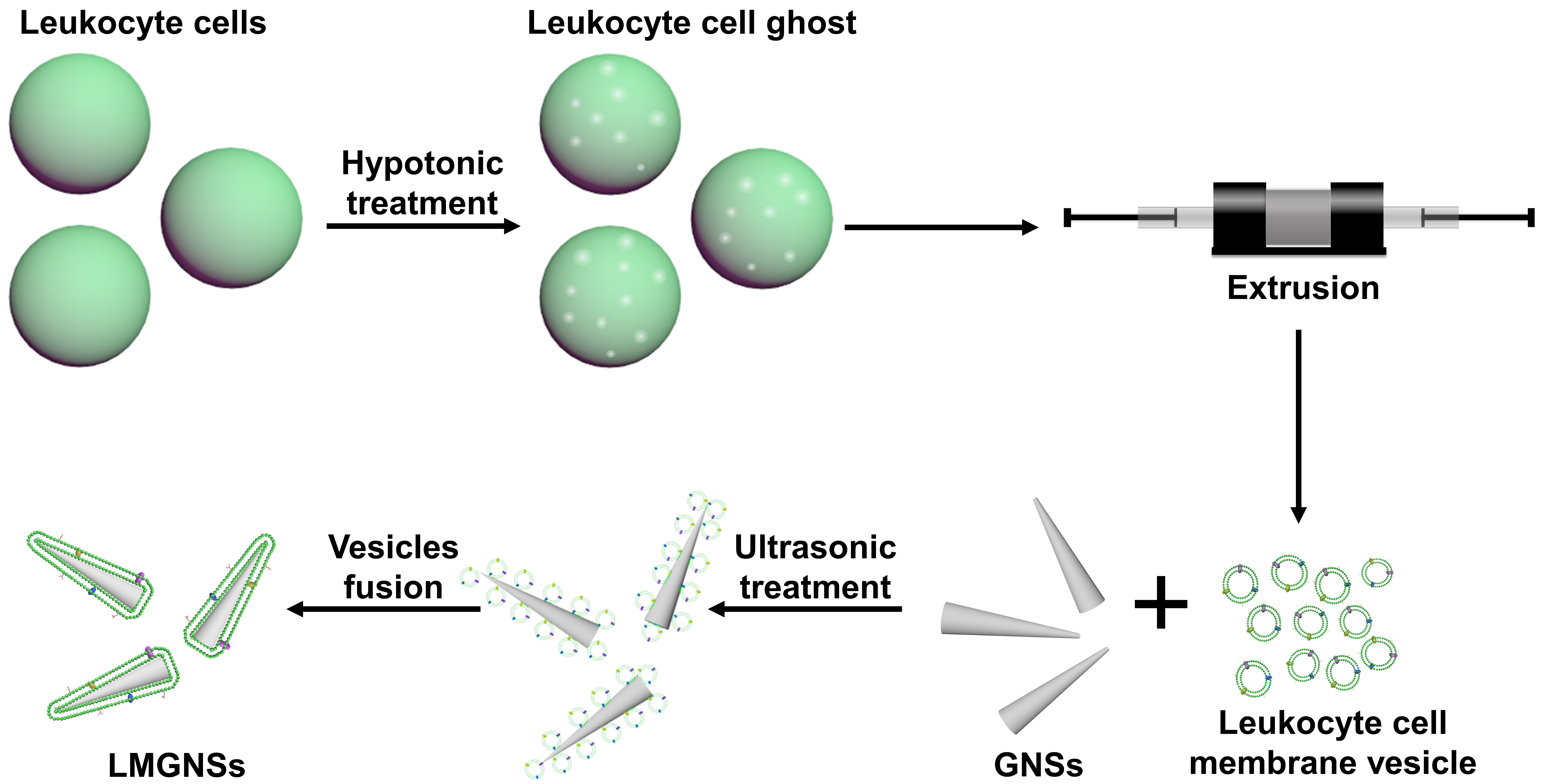


**Figure S3**. The schematic depicting the preparation process of the LMGNSs.


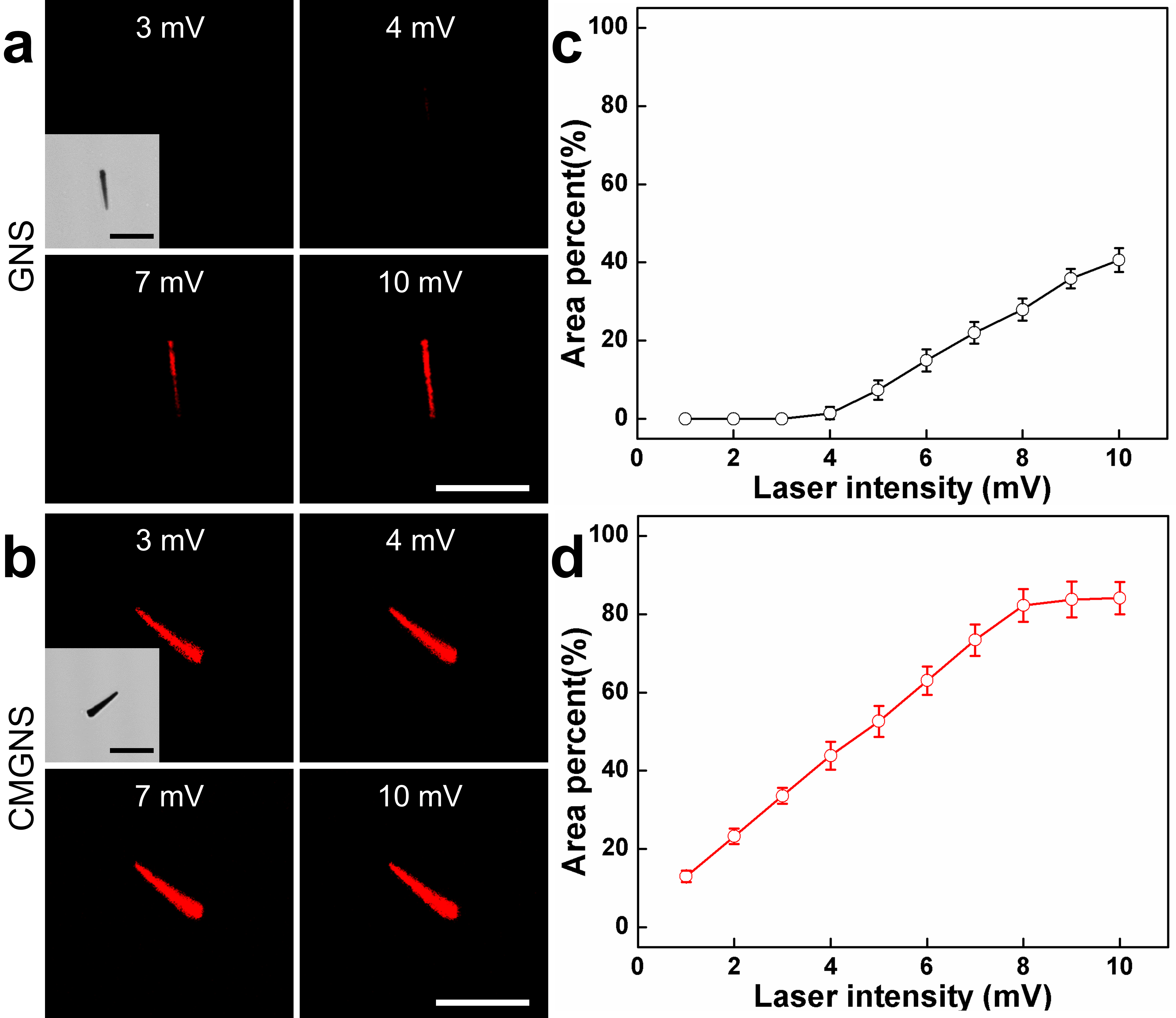


**Figure S4.** The CLSM images of the (a) GNS and (b) LMGNS at a series of laser intensity in 633 nm channel with an acceptable wavelength range from 653 to 673nm. The area percent (the ratio of fluorescent area to bright area) of the (c) GNS and (d) LMGNS. Scale bars, 10 µm.


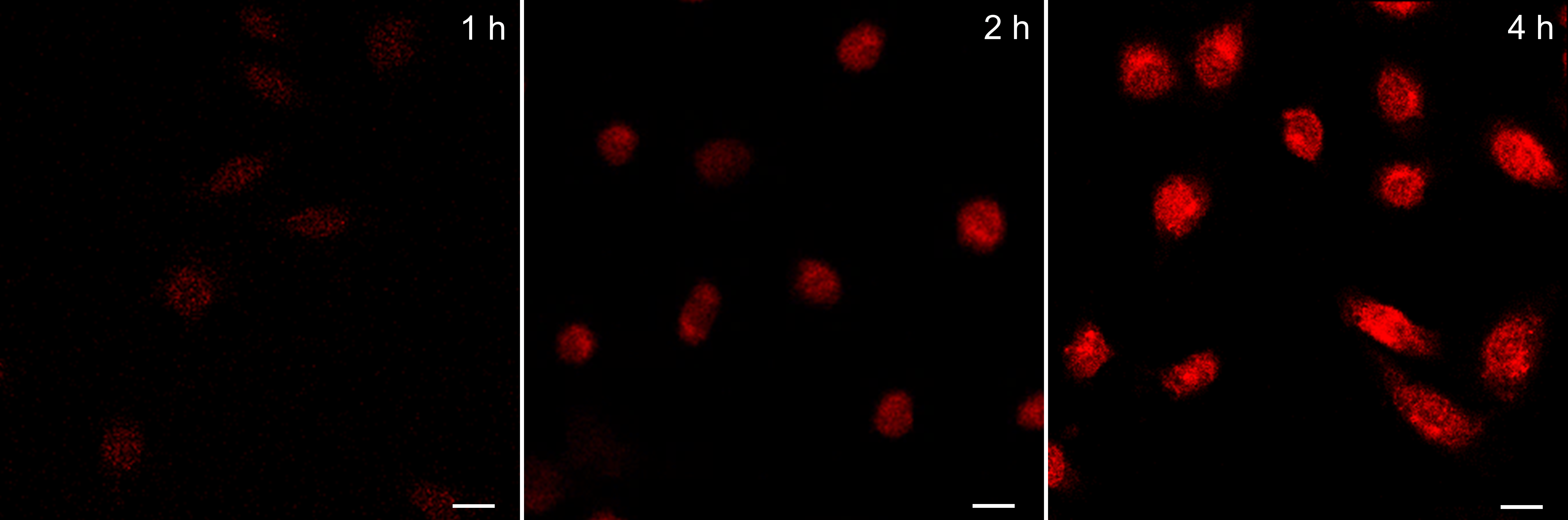


**Figure S5.** CLSM images of the HeLa cells treated with D**ox** loaded GNSs for 1**,** 2 and 4 h. Scale bars, 20 µm.


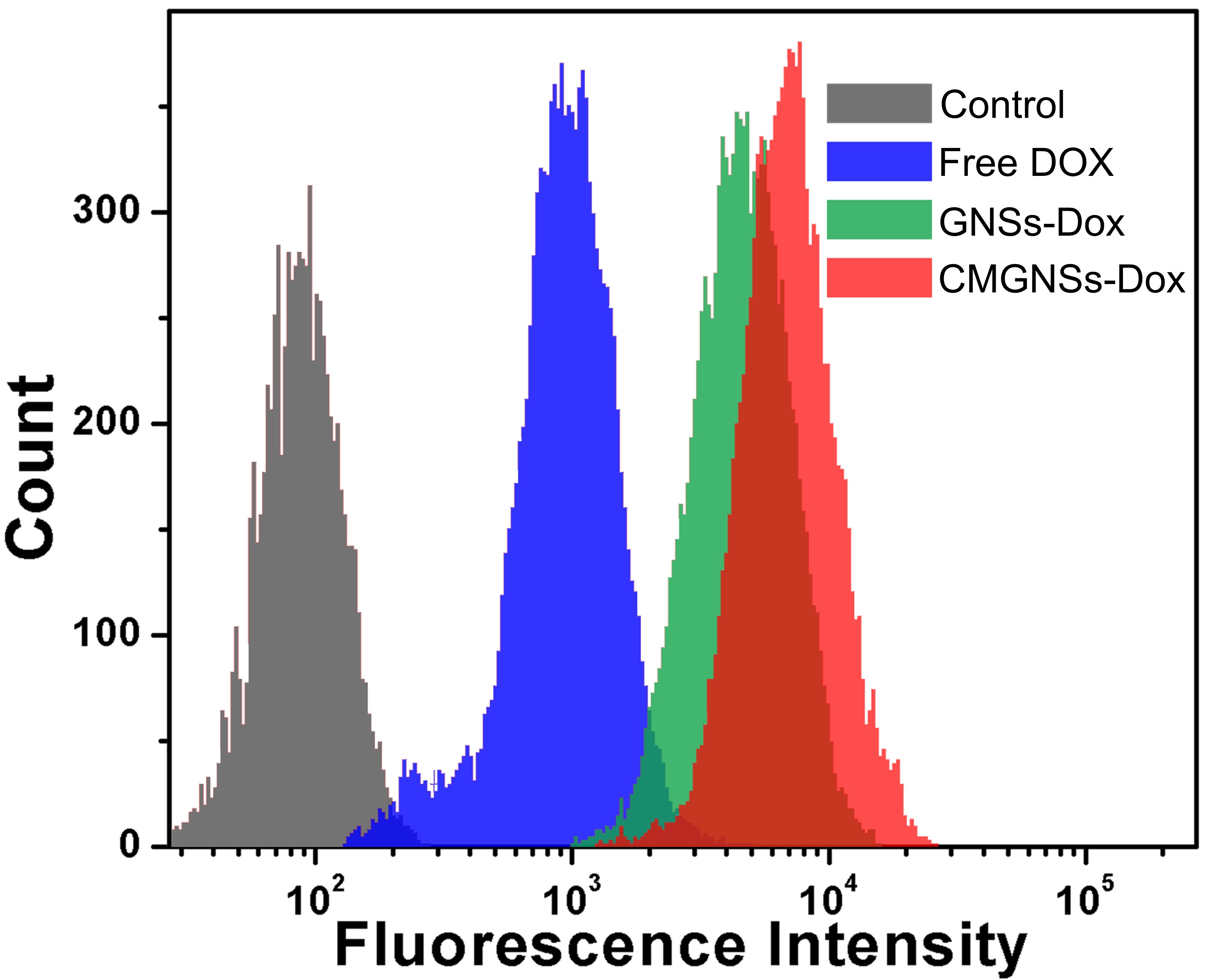


**Fig. S6.** Quantitative Dox release analysis of the control, free Dox, GNSs-Dox and LMGNSs-Dox treatment**s** for **1** h by flow cytometry.
